# Association between number of teeth, dental prostheses, and self-reported dysphagia in brazilian old people: a population-based study

**DOI:** 10.1590/2317-1782/20242023072en

**Published:** 2024-06-21

**Authors:** Marina de Macedo Aquino, Rafaela Soares Rech, Alexandre Baumgarten, Bárbara Niegia Garcia de Goulart

**Affiliations:** 1 Programa de Pós-graduação em Epidemiologia, Faculdade de Medicina,Universidade Federal do Rio Grande do Sul – UFRGS - Porto Alegre (RS), Brasil.; 2 Departamento de Fonoaudiologia, Universidade Federal de Ciências da Saúde de Porto Alegre – UFCSPA - Porto Alegre (RS), Brasil.; 3 Pesquisador independente - Porto Alegre (RS), Brasil.; 4 Curso de Fonoaudiologia, Instituto de Psicologia, Serviço Social e Saúde e Comunicação Humana. Universidade Federal do Rio Grande do Sul – UFRGS, Porto Alegre (RS), Brasil.

**Keywords:** Dysphagia, Number of Teeth, Elderly, Swallowing, Permanent Teeth

## Abstract

**Purpose:**

To investigate the association between the number of permanent teeth and the use of removable dental prostheses with self-reported dysphagia occurrence in individuals aged 60 years or older.

**Methods:**

A population-based cross-sectional study was conducted with 5,432 old individuals who participated in the baseline of the Brazilian Longitudinal Study of Elderly Health (ELSI-Brazil). The outcome "dysphagia" was associated with the number of permanent teeth and the use of removable dental prostheses. Sociodemographic independent variables (age, sex, and race/ethnicity) and clinical history variables (no morbidity, one morbidity, or more than two morbidities) were analyzed using Poisson Regression with robust variance and their respective 95% confidence intervals (CI).

**Results:**

The prevalence of self-reported dysphagia in non-institutionalized old individuals was 30%. The group of old individuals with 10 – 19 natural teeth showed a 52% increased risk of self-reported dysphagia complaint (PRadj 1,565 IC95% 1,34;1,826) compared to their counterparts with more teeth.

**Conclusion:**

An association was found between a lower number of teeth and removable prostheses with the occurrence of dysphagia.

## INTRODUCTION

The stomatognathic system enables the performance of several functions that are essential for the survival and for the health maintenance of individuals, one of them being deglutition^(1)^. Dysphagia is characterized by the difficulty or inability to either form or safely move the food bolus from the mouth to the esophagus^(2)^. Any type or stage of alteration in the deglutition process is considered dysphagia. Health problems directly associated with dysphagia may include malnutrition, dehydration, aspiration pneumonia, and deterioration of the quality of life^(3)^. Premature mortality is the worst indirect outcome of this condition^(4)^.

Older people are particularly susceptible to age-related changes, which can be exacerbated by an unhealthy lifestyle^(5)^. The prevalence of problems with mastication and subsequent difficulty swallowing^(6)^ of food varies among different segments of the older population. This includes those who use dental prostheses, those who need them but do not use them, and those who do not need them^(7)^. This variation highlights the complex nature of the issue and the need for tailored solutions. It should be noted that edentulism, characterized by the absence of natural dentition^(8)^ and the lack of dental prostheses, can impact people's lives with both psychosocial and functional consequences, such as masticatory impairments^(9)^. According to a study on the effects of tooth loss and the dental prosthetic rehabilitation of older patients' deglutition, the prevalence of self-perceived need for a full dental prosthesis was higher among those who needed it and were displeased with their oral health^(10)^. In the Brazilian context, oral rehabilitation with dental prostheses is a national policy priority, due to the high occurrence of edentulism and the low adherence of prosthesis use among older people^(11)^.

Furthermore, the senescence of orofacial functions is known to progress to reduced sensitivity, loss of muscle strength, decreased peristalsis, oral motor impairments and low salivary flow, as well as reduced mobility of the lip, tongue, jaw and face muscles^(12)^.

Therefore, studying the occurrence of dysphagia in the geriatric population, listing their self-perceptions and associated factors is essential to endorse the appropriate and timely care, contributing to a healthy aging and to the quality of life.

Thus, the objective of the present study was to analyze the relationship between the number of teeth, permanent or with the use of removable dental prosthesis, associated with self-reported dysphagia in non-institutionalized Brazilian older people.

## METHOD

This is a population-based cross-sectional study applying a baseline data from the 2015-2016 ELSI-Brazil cohort study. ELSI-Brazil is a longitudinal, household survey conducted in a representative national sample of the population aged 50 years or older^(13)^.

Data collection was done through face-to-face interviews. Trained interviewers went to the participants' homes and administered an individual questionnaire.

ELSI-Brazil was approved by the Ethics Committee of the Fundação Oswaldo Cruz (Oswaldo Cruz Foundation) and the process is registered in Plataforma Brasil (Brazil Platform) (CAAE: 34649814.3.0000.5091). The work presented herein uses anonymous public open data and does not require the use of a free and informed consent form, according to the Brazilian National Council of Health (CNS - Conselho Nacional de Saúde) resolution No. 466/12.

In order to proceed with the sample selection, data from the Demographic Census carried out in 2010 by the Instituto Brasileiro de Geografia e Estatística (IBGE - the Brazilian Institute of Geography and Statistics) were used. 9400 people participated in the ELSI-Brazil's individual questionnaire, located in 70 municipalities of the 5 macro-regions of Brazil.

As an eligibility criterion, were considered individuals who answered the question "In the past 6 months, have you had any difficulty eating or have you felt pain when drinking cold or hot liquids?". Individuals aged 60 years or older who answered the question "In the past 6 months, have you had any difficulty eating or have you felt pain when drinking cold or hot liquids?" were included in the present study. Hence, the effectively eligible sample for the study presented herein was composed of 5432 participants.

The outcome – dysphagia – was characterized as per the following self-reported question: “in the past six months, have you had any difficulty eating or have you felt pain when drinking cold or hot liquids?” response taken from the ELSI-Brazil's individual questionnaire. This question allows for the ensuing answer options: no, yes or unsure/did not answer. The outcome was dichotomized into: no and yes. Unsure/did not answer was classified as missing.

Regarding the exposure variables, the subsequent self-reported Oral Health questions were considered: 1) How many natural teeth do you have left? 0, 1 – 9 teeth, 10 – 19 teeth, 20 teeth or more, or unsure/did not answer. 2) How many natural teeth do you have left on your upper jaw? 0 – 5 teeth, 6 – 11 teeth, 12 teeth and unsure/did not answer. 3) How many natural teeth do you have left on your lower jaw? 0 – 5 teeth, 6 – 11 teeth, 12 teeth and unsure/did not answer. 4) Do you use any type of removable dental prosthesis (artificial denture) to replace the teeth of your lower jaw? No, yes or unsure/did not answer. 4) Do you use any type of removable dental prosthesis (artificial denture) to replace the teeth of your upper jaw? No, yes or unsure/did not answer.” The answers “unsure/did not answer” were recategorized as missing in all questions.

Two sets of adjustment variables were considered: sociodemographic characteristics: sex (female and male), age (60 – 69 years, 70 – 79 years, 80 – 89 years, ≥90), self-reported color/race (white, brown, black, yellow/indigenous). “Unsure/did not answer” responses were categorized as missing.

Clinical history was assessed by stating the following diseases: Heart failure, hypertension, diabetes, stroke, arthritis or rheumatism, osteoporosis, Parkinson's disease, Alzheimer's disease. Subsequently categorized as: no disease, 1 disease, 2 or more diseases. “Unsure/did not answer” responses were categorized as missing.

Absolute and relative frequency analyses were performed with a 95% confidence interval (CI) stratified by the outcome “dysphagia” and Pearson's Chi-square test. To verify the association, crude and adjusted prevalence ratios were performed for the independent variables using Poisson Regression with robust variance with their respective 95% confidence intervals. Data were analyzed using the SPSS v.21 software (Chicago: SPSS Inc).

## RESULTS

Of the 5,432 older participants of the present study, 1,641 (30.2%) had self-reported dysphagia. The group of older patients with 10 to 19 permanent teeth presented a prevalence of (52%) for dysphagia. Most older people with dysphagia are in the 60 to 69 years (58.5%) age group and are self-reportedly black (60.2%). Dysphagia is shown to be as prevalent in men as in women (Table 1).

**Table 1:** Sociodemographic characteristics and clinical history of the older participants in relation to the dysphagia outcome

Dysphagia							
	Total		Yes		No		χ^2^
	n 5428	% 100	n 1641	% 30.2	n 3787	% 69.8	
Age							
60 to 69 years	2,875	53.0	960	58.5	1,915	50.6	<0.001
70 to79 years	1,778	32.8	493	30.0	1,285	33.9	-
> 80 years	775	14.3	188	11.5	587	15.5	3.3
Sex							
Female	3,260	60,0	975	29,9	2,281	70.0	-
Male	2,172	40,0	666	30,7	1,506	69.3	0.319
Color/Race							
White	2,152	42.8	607	39.8	1,545	44.2	-
Black	2,872	57.2	920	60.2	1,952	55.8	8.517
Multimorbidities							
No disease	1,157	21.3	305	26.4	852	73.6	-
1 disease	1927	35.5	533	27.7	1,394	72.3	0.616
>2 disease	2,344	43.1	803	34.3	1,541	65.7	22.328

Older people with 10 to 19 permanent teeth had a higher occurrence of dysphagia (37.3%) even after adjusting for confounding variables. Moreover, regarding older patients with 6 to 11 permanent teeth, there was an occurrence of 37.4% for the upper jaw and 36.8% for the lower jaw, disregarding the use of the prosthesis. When considering prosthesis use, the greater the number of teeth, the less dysphagia (Table 2).

**Table 2:** Chi-square prevalence of oral health of the older people data in relation to the self-reported dysphagia outcome

Dysphagia								
	Total	(%)	Yes	(%)		No	(%)	χ^2^
Main reason for your last dentist appointment	5,205		1,586	30.5		3,619	69.5	
Revision, prevention or check-up	1,056	20.3	253	24.0		803	76.0	<0.001
Pain	346	6.6	123	35.5		223	64.5	-
Extraction	1,576	30.3	528	33.5		1,047	66.4	0.471
Treatment	1,443	27.7	469	32.5		973	67.4	0.283
Other	784	15.1	213	27.2		570	72.7	0.005
How many permanent teeth do you have left	5,279		1,601	30.3		3,678	69.7	
Over 20	878	16.6	234	26.7		644	73.3	-
10 to 19	710	13.4	265	37.3		445	62.7	<0.001
1 to 9	1,337	25.3	486	36.4		851	63.6	<0.001
None	2,354	44.6	616	26.2		1,736	73.7	0.791
How many permanent teeth are left in your upper jaw	5,245		1,590	30.3		3,655	69.7	
Over 12 teeth	690	13.2	173	25.1		517	74.9	<0.001
6 to 11 teeth	503	9.6	188	37.4		315	62.6	-
0 to 5 teeth	4,052	77.2	1,229	30.3		2,821	69.6	0.001
How many permanent teeth are left in your upper jaw	5,351		1,593	29.8		3,758	70.2	
Over 12 teeth	831	15,8	213	25.6		618	74.4	<0.001
6 to 11 teeth	1,189	22.6	438	36.8		751	63.2	-
0 to 5 teeth	3,231	61.5	942	29.2		2,287	70.8	<0.001
Use of some type of removable dental prosthesis in the lower jaw	5,429		1,641	30.2		3,788	69.8	
Yes	2,526	46.5	690	27.3		1,835	72.6	-
No	2,903	53.5	951	32.8		1,949	67.10	<0.001
Use of some type of removable dental prosthesis in the upper jaw	5,430		1,640	30.2		3,790	69.8	
Yes	3,782	69.6	1,094	28.9		2,686	71.00	9.727
No	1,648	30.3	546	33.1		1,100	66.70	-

Among the category with 0 – 5 permanent teeth, both in the upper and lower jaw, most of the participants used removable dentures. Pertaining to the upper jaw, 80.2% of the participants already used prosthesis, as for the lower jaw, this percentage of use drops to 61.1% (Table 3).

**Table 3:** Number of permanent teeth in the upper and lower jaw, in relation to the use of removable dental prosthesis (in the upper and/or lower jaw)

		**Uses Dental Prosthesis**						
		**Total**		**No**			**Yes**	
Permanent teeth left in the upper jaw		n 5,243		n 1,544	% 29.4		n 3,699	% 70.6
0 to 5 teeth		4,050		800	19.8		3,250	80.2
6 to 11 teeth		503		244	48.5		259	51.5
Over 12 teeth		690		500	72.5		190	27.5
Permanent teeth left in the lower jaw		n 5,248		n 2,755	% 52.5		n 2,493	% 47.5
0 to 5 teeth		3,230		1,258	38.9		1,972	61.1
6 to 11 teeth		1,189		812	68.3		377	31.7
Over 12 teeth		829		685	82.6		144	17.4

From the multivariable analysis, adjusted for sociodemographic variables and clinical history, it can be concluded that the more teeth present in the mouth (both permanent and with the use of removable dental prosthesis), the more protected the older people in the sample. It is observed that the group with 10 – 19 permanent teeth, also considering the use of removable dentures, have a lower occurrence of difficulty swallowing (PRadj 1.565 95%CI 1.34;1.826), furthermore, the participants with two or more diseases had a higher prevalence of dysphagia, with p= < 0.001. (Table 4).

**Table 4:** Crude and adjusted analyses* (Poisson regression with robust variance), according to the self-reported dysphagia outcome

		**Crude PR (95% CI)**	**p-value**	χ^2^		**Adjusted PR** ^*^ **(95% CI)**	**p-value**
Age							
>80		0.835 (0.687;1.014)	0.072	0.068		0.840 (0.717;0.983)	0.03
70 – 79		1	-	-		1	-
60 – 69		1.307 (1.148;1.487)	<0.001	<0.001		1.206 (1.095;1.328)	<0.001
**Self-Reported Color/Race**							
White		1	-	-		1	-
Black		1.163 (1.062;1.273)	0.001	0.001		0.956 (0.878;1.041)	0.301
**Sex**							
Female		1	-	-		1	-
Male		1.035 (0.919;1.164)	0.587	0.572		1.017 (0.93;1.112)	0.717
**Multimorbidity**							
None		1	-	-		1	-
1 disease		1.068 (0.906;1.259)	0.550	0.433		1.069 (0.94;1.216)	0.307
> 2 diseases		1.456 (1.245;1.702)	0.229	< 0.001		1.409 (1.25;1.589)	<0.001
**Removable Prosthesis**							
No		1	-	-		1	-
Yes		1.272 (1.120;1.445)	< 0.001	<0.001		0.811 (0.735;0.896)	<0.001
**No. of permanent teeth**							
> 20		1	-	-		1	-
10 to 19		1.639 (1.324;2.029)	< 0.001	< 0.001		1.565 (1.34;1.826)	<0.001
1 to 9		1.572 (1.304;1.894)	< 0.001	< 0.001		1.547 (1.338;1.788)	<0.001
None		0.977 (0.819;1.164)	0.788	0.791		1.182 (1.018;1.373)	0.028
*Adjusted for sociodemographic variables (gender, age, color/race) and clinical history							

## DISCUSSION

The findings of this population-based study with Brazilian older people indicate that there is an association between the number of teeth, whether permanent or due to the use of removable dentures, and the occurrence of self-reported dysphagia in the non-institutionalized older population. The more teeth in the mouth, the lower the incidence of self-reported dysphagia.

The relationships between the number of permanent teeth, the use of dental prostheses and dysphagia in older people can be explained by anatomophysiological hypotheses. The presence of either a reduced number of teeth or their absence may compromise the masticatory efficiency, resulting in problems related to preparing food for proper deglutition. The use of dental prostheses can facilitate mastication. In addition, edentulism can decrease muscle stimulation in the face and mouth, affecting both the strength and coordination of the muscles involved in swallowing.

The precariousness of oral health among older people represents a major public health problem^(14)^. The negative impact of precarious dental care on daily life is particularly significant among individuals with tooth loss, as an effort to reduce masticatory performance, directly affecting the food selection, interfering in the nutritional status and in deglutition^(15)^. Thus, the scientific community has shown an increasing interest in the possible significant associations between oral conditions and systemic outcomes^(16)^.

Hypotheses regarding the motivation behind the scheduling of medical appointments may vary significantly in developing countries, which is the case of Brazil, such as dentist appointments made mostly due to pain. While other reasons remain common, like routine checkups, fillings or other further treatments for dental cavities and pain, there are still some remaining challenges to address due to the lack of regular access to preventive dentistry^(17)^.

Chronic diseases, obesity and hypertension are recurrent in older people, revealing conditions that be detrimental to healthy aging^(18)^. The high disease burden in the older population is an issue that seems to be closely associated with the deglutition process, as presented herein.

Older people belonging to black and brown ethnic groups, who are dichotomized as black, have a greater propensity to develop diseases when compared to other ethnic groups. This disparity can be attributed, to a certain extent, to the socioeconomic factors and the quality of life of these individuals^(19)^.

Socioeconomic inequality, which encompasses limited access to educational resources, quality employment, qualified health services, and adequate housing, can lead to unfavorable living conditions, such as poor housing environment, inadequate nutrition, and less access to preventive medical care. These unfavorable socioeconomic factors have been associated with an increased risk of developing chronic disorders among the older population, such as diabetes, hypertension and cardiovascular diseases, as discussed in the article^(20)^.

The epidemiological panorama indicates that these individuals have survived with multiple coexisting health problems and that aging is strongly correlated to the population's reduced capacity^(21)^. It is a well-established fact that senescence promotes the development of vulnerabilities and the propensity to develop diseases^(22)^. Therefore, it is important to highlight that dysphagia in the Brazilian older people is subject to chronic diseases.

The association found with multimorbidity suggests that dysphagia, a disorder that involves real or self-perceived difficulty to either form or safely move a bolus from the oral cavity to the esophagus, should be seen as a symptom of many pathological processes^(23)^.

This condition has been considered a “geriatric syndrome”, since the older population with multimorbidities have a greater susceptibility to dysphagia, due to the significant demand that the deglutition imposes on the swallowing process^(24)^. Deglutition involves a complex coordination of oropharyngeal muscles and structures, and any alteration in this process can lead to difficulties in the food bolus' formation and safe transportation^(25)^.

The less aged older people tend to show more unfamiliarity regarding the identification of self-perceived dysphagia, while the older individuals have an increased risk of mortality, the youngest-old are more susceptible to developing morbidities. These findings indicate that age is a critical factor to be considered when assessing the health and well-being of older people^(26)^.

Insufficient epidemiological surveys with the appropriate methodological rigor along with representative samples of the population express the prevalence of alterations in deglutition among the older population^(27)^. Convenience samples are common, and the inferences represent only the disease prevalence in a specific population^(28,29)^. Thus, the greatest strength of the present study is to have a population-based, non-institutionalized sample, which contributes to the representativeness and generalizability of the results, reflecting different profiles of the older people in the community. Additionally, the availability of information on various socioeconomic, behavioral circumstances, health and oral care status allowed for a more insightful investigation on different relevant concerns related to the aging process, such as: race, age, sex, multimorbidities, number of teeth and dysphagia.

## STUDY LIMITATIONS

The present study analyzed the perception of dysphagia through self-reported data, but not through a clinical evaluation of this condition. Instruments for screening, as well as for the clinical assessment or complementary diagnostic of dysphagia could attest the most accurate prevalence regarding this population. Nevertheless, self-perception is an indicator that has been shown to be relevant for several diseases and, in this case, it provides an overview of the prevalence of dysphagia at population-level, concerning the non-institutionalized older people, which could hardly be evaluated using specific and specialized exams or screening^(30)^. Furthermore, the self-perception of an affliction such as dysphagia, which significantly impacts the individual's dietary choices, is considered herein to be a culturally relevant social activity as well as a daily routine in our society, presenting a high accuracy rate, possibly even higher than clinical examinations, which are greatly influenced by evaluator bias. The period of the data collection is not deemed to be a limiting factor, since the application of consistent and methodologically robust data can offer a valuable basis for several purposes in research, albeit being collected a few years ago. This approach enables historical comparisons, the analysis of changes over time in specific variables and provides a solid foundation for longitudinal studies. Moreover, this data can serve as a platform to generate hypotheses and direct future investigations, identifying areas that require further appraisal.

It is equally important to emphasize that this research used pre-COVID-19 pandemic data. Post-pandemic data is currently available and the next stage of this study, which is already in progress, is precisely designed to evaluate dysphagia in the post-covid scenario, taking the surviving population into consideration.

## CONCLUSION

The findings of the present study demonstrate that the lower the number of teeth, permanent or with the use of removable dentures, the more prevalent the self-reported dysphagia in non-institutionalized Brazilian older people. These results corroborate the need to further study the attributable fraction of the number of teeth, their location in the dental arch and their role in the stomatognathic system at population level, in this age group, in relation to the occurrence of dysphagia.
