# Supplementary material for: Association between number of teeth, dental prostheses, and self-reported dysphagia in brazilian old people: a population-based study
Source: Codas. 2024 Jun 21;36(4):e20230072. doi: 10.1590/2317-1782/20242023072en (PMC11296669; doi:10.1590/2317-1782/20242023072en)
Supplement: Translation

# Translation: Associação entre número de dentes, próteses dentárias e disfagia autorreferida em idosos brasileiros: estudo populacional

**DOI:** 10.1590/2317-1782/20242023072pt

**Authors:** Marina de Macedo Aquino, Rafaela Soares Rech, Alexandre Baumgarten, Bárbara Niegia Garcia de Goulart

**Affiliations:** 1 Programa de Pós-graduação em Epidemiologia, Faculdade de Medicina,Universidade Federal do Rio Grande do Sul – UFRGS - Porto Alegre (RS), Brasil.; 2 Departamento de Fonoaudiologia, Universidade Federal de Ciências da Saúde de Porto Alegre – UFCSPA - Porto Alegre (RS), Brasil.; 3 Pesquisador independente - Porto Alegre (RS), Brasil.; 4 Curso de Fonoaudiologia, Instituto de Psicologia, Serviço Social e Saúde e Comunicação Humana. Universidade Federal do Rio Grande do Sul – UFRGS, Porto Alegre (RS), Brasil.

**Keywords:** Disfagia, Número de Dentes, Idoso, Deglutição, Dentes Permanentes

## Abstract

**Objetivo:**

Verificar a associação entre o número de dentes e uso de prótese dentária removível e a ocorrência de disfagia autorreferida em idosos de 60 anos ou mais.

**Método:**

Estudo transversal de base populacional com 5.432 idosos, que participaram da linha de base do Estudo Longitudinal da Saúde do Idoso (ELSI-Brasil). O desfecho “disfagia” foi associado ao número de dentes permanentes e ao uso de prótese dentária removível. As variáveis independentes sociodemográficas (idade, sexo e cor/raça) e de histórico clínico (nenhuma morbidade, uma morbidade ou mais de duas morbidades) utilizando Regressão de Poisson com variância robusta e seus respectivos intervalos de confiança (IC) de 95% foram analisados.

**Resultados:**

A prevalência de disfagia autorreferida nos idosos não institucionalizados foi de 30%. O grupo de idosos com 10 – 19 dentes permanentes apresentou um risco de 52% de queixa de disfagia autorreferida (RPaj 1,565 IC95% 1,34;1,826) se comparado com seus pares com mais dentes.

**Conclusão:**

foi encontrada associação entre o menor número de dentes e próteses removíveis com a ocorrência de disfagia.

## INTRODUÇÃO

O sistema estomatognático possibilita a realização de funções essenciais para a sobrevivência e a manutenção da saúde, entre elas a deglutição^(1)^. A disfagia se caracteriza pela dificuldade ou incapacidade de formar ou mover o bolo alimentar com segurança da boca ao esôfago^(2)^, qualquer tipo ou grau de alteração no processo de deglutição é considerada disfagia. Os problemas de saúde diretamente relacionados à disfagia incluem desnutrição, desidratação, pneumonia aspirativa, e piora na qualidade de vida^(3)^. A mortalidade precoce é a pior consequência indireta relacionada a esta condição^(4)^.

A população idosa sofre com as alterações decorrentes do envelhecimento e estas são agravadas por condições de estilo de vida não saudáveis^(5)^. A prevalência de dificuldades na mastigação e consequentes prejuízos na deglutição^(6)^ dos alimentos variam entre os idosos que utilizam próteses, os que não utilizam, mas necessitam, e aqueles que não têm a necessidade de uso de prótese^(7)^.

Cabe destacar que o edentulismo, caracterizado pela ausência de dentes permanentes na boca^(8)^, e a falta de próteses dentárias, impactam psicossocial e funcionalmente a vida das pessoas, incluindo dificuldades de mastigação^(9)^. Estudo sobre o impacto da perda de dentes e a reabilitação oral protética na deglutição em idosos revelou que a autopercepção da necessidade de prótese total foi mais prevalente entre aqueles que necessitavam de prótese dentária e estavam insatisfeitos com a sua saúde bucal^(10)^. No contexto brasileiro, a reabilitação oral com próteses dentárias é uma prioridade da política nacional devido à alta frequência de perda de dentes e ao baixo uso de próteses entre os idosos^(11)^.

Ademais, sabe-se que a senescência das funções orofaciais resulta em diminuição de sensibilidade, força muscular, velocidade de contração, capacidade motora oral e fluxo salivar, assim como na redução no movimento dos lábios, língua, mandíbula e músculos da face^(12)^.

Diante deste cenário, estudar a distribuição da disfagia na população geriátrica, elencando as suas autopercepções e os fatores associados é essencial para embasar um cuidado apropriado e em tempo oportuno, contribuindo para um envelhecimento saudável e com qualidade de vida.

Assim, o objetivo deste estudo foi analisar a relação entre o número de dentes, permanentes ou com uso de prótese dentária removível, associados à disfagia autorreferida em idosos brasileiros não institucionalizados.

## MÉTODO

Trata-se de um estudo transversal de base populacional, baseado nos dados da linha de base do estudo de coorte do ELSI-Brasil nos anos de 2015-2016. ELSI-Brasil é uma pesquisa longitudinal, de base domiciliar, conduzida em amostra nacional representativa da população com 50 anos ou mais^(13)^.

A coleta de dados foi feita por meio de entrevistas presenciais. Entrevistadores treinados foram até a residência dos participantes, e aplicaram um questionário individual.

O ELSI-Brasil foi aprovado pelo Comitê de Ética da Fundação Oswaldo Cruz e o processo está cadastrado na Plataforma Brasil (CAAE: 34649814.3.0000.5091). O trabalho ora apresentado faz uso de dados públicos abertos anônimos e não demanda uso de termo de consentimento livre e esclarecido, conforme resolução CNS 466/12.

A pesquisa foi aprovada pelo Comitê de Ética e Pesquisa (CEP) do Centro de Minas Gerais e o processo está cadastrado na Plataforma Brasil (Protocolo no. 886.754).

Para a definição da amostra foram utilizados dados do Censo Demográfico realizado pelo Instituto Brasileiro de Geografia e Estatística (IBGE), no ano de 2010. Participaram do questionário individual do ELSI-Brasil, 9400 pessoas, localizadas em 70 municípios das 5 macrorregiões do Brasil.

Como critério de inclusão, foram considerados indivíduos que responderam à questão “Nos últimos 6 meses, o senhor(a) teve dificuldade para comer ou sentiu dor ao tomar líquidos gelados ou quentes?” Foram incluídos no estudo os indivíduos com 60 anos ou mais que responderam à questão “Nos últimos 6 meses, o senhor(a) teve dificuldade para comer ou sentiu dor ao tomar líquidos gelados ou quentes?”. Portanto, a amostra efetivamente elegível para o estudo apresentado foi composta por 5432 participantes.

O desfecho – disfagia – foi criado a partir da seguinte questão autorreferida: “nos últimos seis meses, teve dificuldade para comer ou sentiu dor ao tomar líquidos gelados ou quentes?” resposta retirada do questionário individual do ELSI-Brasil. Esta questão oferece opções de resposta: não, sim ou não sabe/ não respondeu. O desfecho foi dicotomizado em: não e sim. Não sabe/não respondeu foi classificado como *missing*.

Para variáveis de exposição foram consideradas as seguintes questões autorrelatadas de Saúde Bucal: 1) Quantos dentes naturais o senhor(a) tem? 0, 1 – 9 dentes, 10 – 19 dentes, 20 dentes ou mais, ou não sabe/não respondeu. 2)Quantos dentes naturais o senhor(a) tem na parte de cima da boca? 0 – 5 dentes, 6 – 11 dentes, 12 dentes e Não sabe/não respondeu. 3)Quantos dentes naturais? o senhor(a) tem na parte de baixo da boca? 0 – 5 dentes, 6 – 11 dentes, 12 dentes e não sabe/não respondeu. 4)O senhor(a) usa algum tipo de prótese dentária removível (dente artificial) para substituir os dentes na parte de baixo da boca? não, sim ou não sabe/não respondeu. 5) O senhor(a) usa algum tipo de prótese dentária removível (dente artificial) para substituir os dentes na parte de cima da boca? não, sim ou não sabe/não respondeu.” As respostas “não sabe/não respondeu” foram recategorizadas como *missing* em todas as perguntas.

Foram considerados dois grupos de variáveis de ajuste: características sociodemográficas: sexo (feminino e masculino), idade (60 – 69 anos, 70 – 79 anos, 80 – 89 anos, ≥90), autorrelato de cor/raça (branca, parda, preta, amarela/indígena). As respostas “não sabe/não respondeu” foram categorizadas como *missing*.

O histórico clínico foi avaliado por meio da afirmação das seguintes doenças: Insuficiência cardíaca, hipertensão arterial, diabetes, acidente vascular encefálico, artrite ou reumatismo, osteoporose, doença de Parkinson, doença de Alzheimer. Posteriormente categorizadas como: nenhuma doença, 1 doença, 2 ou mais doenças. As respostas “não sabe/não respondeu” foram categorizadas como *missing*.

Foram realizadas análises de frequência absoluta e relativa com intervalo de confiança (IC) de 95% estratificadas pelo desfecho “disfagia” e Teste Qui-quadrado de Pearson. Para verificar a associação foram realizadas razões de prevalências brutas e ajustadas para as variáveis independentes utilizando Regressão de Poisson com variância robusta com seus respectivos intervalos de confiança de 95%. Os dados foram analisados usando o software SPSS v.21 (Chicago: SPSS Inc).

## RESULTADOS

Dos 5.432 idosos participantes do estudo, 1.641 (30,2%) autorreferiram disfagia. O grupo de idosos com 10 a 19 dentes permanentes apresentaram prevalência de (52%) para disfagia. A maioria dos idosos com disfagia está na faixa etária de 60 até 69 anos (58,5%) e negros (60,2%). A disfagia é tão prevalente em homens quanto em mulheres (Tabela 1).

**Tabela 1:** Características sociodemográficas e histórico clínico dos idosos participantes do estudo em relação ao desfecho disfagia

Disfagia							
	Total		Sim		Não		χ^2^
	n 5428	% 100	n 1641	% 30,2	n 3787	% 69,8	
Idade							
De 60 a 69 anos	2.875	53,0	960	58,5	1.915	50,6	<0,001
De 70 a 79 anos	1.778	32,8	493	30,0	1.285	33,9	-
> 80 anos	775	14,3	188	11,5	587	15,5	3,3
Sexo							
Feminino	3.260	60,0	975	29,9	2.281	70,0	-
Masculino	2.172	40,0	666	30,7	1.506	69,3	0,319
Cor/Raça							
Branco	2.152	42,8	607	39,8	1.545	44,2	-
Preto	2.872	57,2	920	60,2	1.952	55,8	8,517
Multimorbidades							
Nenhuma doença	1.157	21,3	305	26,4	852	73,6	-
1 doença	1.927	35,5	533	27,7	1.394	72,3	0,616
>2 doenças	2.344	43,1	803	34,3	1.541	65,7	22,328

Idosos com 10 a 19 dentes permanentes em boca apresentaram maior ocorrência na disfagia (37,3%) ainda após o ajuste de variáveis confundidoras. Já idosos com 6 a 11 dentes permanentes apresentaram ocorrência de 37,4% na arcada superior e 36,8% na arcada inferior, desconsiderando o uso da prótese. Ao considerar o uso da prótese, quanto maior o número de dentes, menos disfagia (Tabela 2).

**Tabela 2:** Prevalência qui-quadrado de dados de saúde bucal dos idosos participantes do estudo em relação ao desfecho disfagia autorreferida

Disfagia								
	Total	(%)	Sim	(%)		Não	(%)	χ^2^
Principal motivo da sua última consulta ao dentista	5.205		1.586	30,5		3.619	69,5	
Revisão, prevenção ou check-up	1.056	20,3	253	24,0		803	76,0	<0,001
Dor	346	6,6	123	35,5		223	64,5	-
Extração	1.576	30,3	528	33,5		1.047	66,4	0,471
Tratamento	1.443	27,7	469	32,5		973	67,4	0,283
Outros	784	15,1	213	27,2		570	72,7	0,005
Quantos dentes permanentes possui na boca	5.279		1.601	30,3		3.678	69,7	
Acima de 20	878	16,6	234	26,7		644	73,3	-
10 a 19	710	13,4	265	37,3		445	62,7	<0,001
1 a 9	1.337	25,3	486	36,4		851	63,6	<0,001
Nenhum	2.354	44,6	616	26,2		1.736	73,7	0,791
Quantos dentes permanentes tem na arcada superior	5.245		1.590	30,3		3.655	69,7	
Acima de 12 dentes	690	13,2	173	25,1		517	74,9	<0,001
De 6 a 11 dentes	503	9,6	188	37,4		315	62,6	-
De 0 a 5 dentes	4.052	77,2	1.229	30,3		2.821	69,6	0,001
Quantos dentes permanentes tem na arcada inferior	5.351		1.593	29,8		3.758	70,2	
Acima de 12 dentes	831	15,8	213	25,6		618	74,4	<0,001
De 6 a 11 dentes	1.189	22,6%	438	36,8%		751	63,2%	-
De 0 a 5 dentes	3.231	61,5%	942	29,2%		2.287	70,8%	<0,001
Usa algum tipo de prótese dentária removível na arcada dentária inferior	5.429		1.641	30,2		3.788	69,8	
Sim	2.526	46,5%	690	27,3%		1.835	72,6%	-
Não	2.903	53,5	951	32,8		1.949	67,10	<0,001
Usa algum tipo de prótese dentária removível na arcada dentária superior	5.430		1.640	30,2		3.790	69,8	
Sim	3.782	69,6	1.094	28,9		2.686	71,00	9,727
Não	1.648	30,3	546	33,1		1.100	66,70	-

A categoria com 0 – 5 dentes permanentes na boca, tanto na arcada superior quanto inferior, fazem uso de próteses dentárias removíveis, em sua maioria. Referente a arcada superior 80,2% usam prótese já na arcada inferior o uso cai para 61,1% (Tabela 3).

**Tabela 3:** Número de dentes permanentes na arcada superior e inferior, em relação ao uso de prótese dentária removível (na arcada superior e/ou inferior)

**Usa Prótese dentária**							
	**Total**		**Não**			**Sim**	
Dentes permanentes na arcada superior	n 5.243		n 1.544	% 29,4		n 3.699	% 70,6
De 0 a 5 dentes	4.050		800	19,8%		3.250	80,2%
De 6 a 11 dentes	503		244	48,5%		259	51,5%
Acima de 12 dentes	690		500	72,5%		190	27,5%
Dentes permanentes na arcada inferior	n 5.248		n 2.755	% 52,5		n 2.493	% 47,5
De 0 a 5 dentes	3.230		1.258	38,9%		1.972	61,1%
De 6 a 11 dentes	1.189		812	68,3%		377	31,7%
Acima de 12 dentes	829		685	82,6%		144	17,4%

A partir da análise multivariável, ajustada para as variáveis sociodemográficas e histórico clínico, observa-se que quanto mais dentes em boca (tanto permanentes quanto com o uso de prótese dentária removível), mais protegidos estão os idosos da amostra. Observa-se que o grupo com 10 – 19 dentes permanentes, e considerando o uso de prótese removível na boca, têm menor ocorrência de dificuldade para engolir (RPaj 1,565 IC95% 1,34;1,826) bem como idosos com duas ou mais doenças apresentaram maior prevalência com disfagia, com o p= < 0,001. (Tabela 4).

**Tabela 4:** Análises bruta e ajustada* (regressão de Poisson com variância robusta), segundo o desfecho disfagia autorreferida

		**RP bruta (IC 95%)**	**p-valor**	χ^2^		**RP ajustada** ^*^ **(IC 95%)**	**p-valor**
**Idade**							
>80		0,835 (0,687;1,014)	0,072	0,068		0,840 (0,717;0,983)	0,03
70 – 79		1	-	-		1	-
60 – 69		1,307 (1,148;1,487)	<0,001	<0,001		1,206 (1,095;1,328)	<0,001
**Autorrelato Cor/Raça**							
Branco		1	-	-		1	-
Negro		1,163 (1,062;1,273)	0,001	0,001		0,956 (0,878;1,041)	0,301
**Sexo**							
Feminino		1	-	-		1	-
Masculino		1,035 (0,919;1,164)	0,587	0,572		1,017 (0,93;1,112)	0,717
**Multimorbidade**							
Nenhum		1	-	-		1	-
1 doença		1,068 (0,906;1,259)	0,550	0,433		1,069 (0,94;1,216)	0,307
> 2 doenças		1,456 (1,245;1,702)	0,229	< 0,001		1,409 (1,25;1,589)	<0,001
**Prótese Removível**							
Não		1	-	-		1	-
Sim		1,272 (1,120;1,445)	< 0,001	<0,001		0,811 (0,735;0,896)	<0,001
**Nº de dentes permanentes**							
> 20		1	-	-		1	-
10 a 19		1,639 (1,324;2,029)	< 0,001	< 0,001		1,565 (1,34;1,826)	<0,001
1 a 9		1,572 (1,304;1,894)	< 0,001	< 0,001		1,547 (1,338;1,788)	<0,001
Nenhum		0,977 (0,819;1,164)	0,788	0,791		1,182 (1,018;1,373)	0,028
*Ajustado para variáveis sociodemográficas (sexo, idade, cor/raça) e histórico clínico							

## DISCUSSÃO

Os achados deste estudo de base populacional com idosos brasileiros indica que há associação entre o número de dentes em boca, sejam permanentes ou por uso de próteses dentárias removíveis, e a ocorrência de disfagia autorreferida em idosos não institucionalizados. Quanto mais dentes em boca, menor a ocorrência de disfagia autorreferida.

As relações entre o número de dentes permanentes, o uso de próteses dentárias e a disfagia em idosos podem ser explicadas por hipóteses anatomofisiológicas. A presença de um menor número de dentes ou ausência deles pode comprometer a eficiência da mastigação, resultando em dificuldades na preparação dos alimentos para a deglutição. O uso de próteses dentárias pode facilitar a mastigação. Além disso, a falta de dentes pode diminuir a estimulação muscular na face e na boca, afetando a força e a coordenação dos músculos envolvidos na deglutição.

A precariedade da saúde bucal entre os idosos representa um importante problema de saúde pública^(14)^. O impacto negativo das precárias condições bucais sobre a vida cotidiana é particularmente significante entre os indivíduos com perda de dentes, a fim de reduzir o desempenho mastigatório, interferindo diretamente na escolha dos alimentos a serem ingeridos, intervir no estado nutricional e no processo de deglutição^(15)^. Deste modo, a comunidade científica tem demonstrado cada vez mais interesse entre possíveis relações entre as condições orais e os desfechos sistêmicos^(16)^.

Hipóteses para visitas ao dentista serem em sua maioria por dor, podem variar significativamente em países em desenvolvimento, que é o caso do Brasil. Enquanto alguns motivos, como exames de rotina, tratamento de cáries e dores dentárias, permanecem comuns, há desafios adicionais devido à falta de acesso regular à odontologia preventiva^(17)^.

Doenças crônicas, obesidade e hipertensão são achados corriqueiros em idosos, revelando condições que prejudicam o envelhecimento saudável^(18)^. A alta carga de doença na população idosa é um desafio que parece estar intimamente associado ao processo de deglutição, como apresentado neste estudo.

Idosos pertencentes aos grupos étnicos pretos e pardos, que são dicotomizados como pretos, apresentam uma maior propensão ao desenvolvimento de doenças quando comparados aos demais grupos étnicos. Essa disparidade pode ser atribuída, em parte, ao fator socioeconômico e à qualidade de vida desses indivíduos^(19)^.

A desigualdade socioeconômica, que engloba acesso limitado a recursos educacionais, emprego de qualidade, serviços de saúde adequados e moradia adequada, pode levar a condições de vida desfavoráveis, tais como condições de habitação precárias, alimentação inadequada e menor acesso a cuidados médicos preventivos. Esses fatores socioeconômicos desfavoráveis têm sido associados ao aumento do risco de desenvolvimento de doenças crônicas, como diabetes, hipertensão arterial e doenças cardiovasculares, entre a população idosa, conforme discutido no artigo^(20)^.

O panorama epidemiológico revela que estes indivíduos têm sobrevivido com múltiplos problemas de saúde coexistentes e que o envelhecimento vem fortemente acompanhado de capacidade reduzida da população^(21)^. Sabe-se que a senescência provoca vulnerabilidade e propensão ao desenvolvimento de doenças^(22)^. Dessa forma, é relevante destacar que a disfagia na população idosa brasileira está sujeita a doenças crônicas.

A associação encontrada com a multimorbidade sugere que a disfagia, condição que envolve a dificuldade real ou autopercebida na formação ou movimentação segura de um bolo da cavidade oral para o esôfago, deve ser vista como um sintoma de muitos processos patológicos^(23)^.

Esta condição tem sido considerada uma “síndrome geriátrica”, idosos com multimorbidades têm uma maior suscetibilidade à disfagia devido à demanda significativa que o ato de engolir impõe ao sistema de deglutição^(24)^. O processo de deglutição envolve uma complexa coordenação de músculos e estruturas orofaríngeas, e qualquer alteração nesse processo pode levar a dificuldades na formação e no transporte seguro do bolo alimentar^(25)^.

Idosos com menos idade tendem a mostrar mais estranhamento ao identificar a disfagia, ao mesmo tempo que, idosos com maior idade têm maior probabilidade de mortalidade, enquanto aqueles mais jovens são mais suscetíveis a desenvolver morbidades. Esses achados sugerem que a idade é um fator crítico a ser considerado ao avaliar a saúde e o bem-estar dos idosos^(26)^.

Insuficientes levantamentos epidemiológicos com rigor metodológico apropriado e amostras representativas da população expressam a prevalência das alterações na deglutição na população idosa^(27)^. As amostras de conveniência são comuns e as inferências representam apenas a frequência deste agravo em uma população específica^(28,29)^. Assim, a maior fortaleza deste estudo é contar com uma amostra de base populacional, não institucionalizada, a qual contribui para a representatividade e generalização dos resultados, os quais refletem diferentes perfis de idosos na comunidade. Além disso, a disponibilidade de informações sobre diversos cenários socioeconômicos, comportamentais, de saúde e de saúde bucal possibilitou o aprofundamento de diferentes e importantes questões no processo de envelhecimento, tais como: cor, idade, sexo, multimorbidades, número de dentes e disfagia.

## LIMITAÇÕES DO ESTUDO

Este estudo analisou a percepção da disfagia por meio de autorrelato, mas não por uma avaliação clínica desta condição. Instrumentos de rastreio, avaliação clínica ou complementar da disfagia poderiam expressar a prevalência mais acurada nesta população. Por outro lado, a autopercepção é um indicador que tem se mostrado relevante para diversos agravos e, neste caso, contribui para ter um panorama da prevalência de disfagia em nível populacional, em idosos, em um quantitativo populacional não institucionalizado, que muito dificilmente seria avaliado com exames ou rastreamentos específicos e especializados^(30)^. Além disso, consideramos que a autopercepção para um agravo como a disfagia, que impacta de forma significativa a alimentação, uma atividade social e de rotina relevantes culturalmente na nossa sociedade, possui uma alta acurácia, possivelmente ainda maior do que exames clínicos, os quais são altamente dependente de viés de avaliador. Não consideramos que a data da coleta seja um fator limitador, visto que o uso de dados consistentes e metodologicamente robustos, mesmo que coletados há alguns anos, pode oferecer uma base valiosa para diversos propósitos na pesquisa. Essa abordagem possibilita comparações históricas, a análise de mudanças ao longo do tempo em variáveis específicas e fornece um fundamento sólido para estudos longitudinais. Além disso, esses dados podem servir como uma plataforma para gerar hipóteses e direcionar investigações futuras, identificando áreas que merecem uma exploração mais aprofundada.

Ressaltamos também, que esses dados são pré-pandemia da COVID 19. Já temos dados do pós-pandemia e a próxima etapa do estudo, já em andamento, é justamente avaliar a disfagia no pós-covid, com os sobreviventes.

## CONCLUSÃO

Os achados deste estudo demonstram que quanto menor o número de dentes, permanentes ou com o uso de próteses removíveis, mais prevalente a disfagia autorreferida em idosos brasileiros não institucionalizados. Esses resultados sustentam a necessidade de estudar com mais detalhes a fração atribuível do número de dentes, sua localização na arcada dentária e o seu papel no conjunto do sistema estomatognático em nível populacional, nessa faixa etária, em relação à ocorrência de disfagia.
